# Emphysematous hepatitis with successful treatments: A rare case report

**DOI:** 10.1097/MD.0000000000032530

**Published:** 2023-01-27

**Authors:** Nannan Pan, Shuo Wang, Zhenwei Miao

**Affiliations:** a Department of Radiology, Tianjin Medical University Baodi Clinical College: Tianjin Baodi Hospital, Tianjin, China; b Department of Orthopaedic Surgery, Tianjin Medical University Baodi Clinical College: Tianjin Baodi Hospital, Tianjin, China.

**Keywords:** case report, computed tomography (CT), diabetes mellitus, effective treatments, emphysematous hepatitis (EH)

## Abstract

Emphysematous hepatitis (EH) is a rare and fulminant gas-forming liver infection. Only 3 patients were successfully treated. Diabetes mellitus and a history of digestive system cancer may predispose individuals to EH. Computed tomography (CT) findings support the diagnosis of EH and monitor progress.

**Patient concerns:**

A 48-year-old man with diabetes presented with nausea, vomiting (gastric contents) and diarrhea. Laboratory test results revealed elevated levels of inflammatory indicators and abnormal liver function. CT showed a large-scale air collection with some remaining parenchymal debris in the left lobe of the liver. Remarkably, no fluid was observed inside the lesion.

**Diagnose:**

The abdominal CT features and laboratory examination results rationalized the diagnosis of EH.

**Interventions and outcomes:**

The patient finally recovered from this severe disease through a series of effective treatments, including strict glucose control, sensitive antibiotic therapy, and subsequent percutaneous drainage.

**Lessons:**

EH generally deteriorates rapidly and eventually leads to death. This case will raise awareness of the rare and severe disease, strengthen diagnostic capacities, and provide advice to treat it.

## 1. Introduction

Emphysematous hepatitis (EH) is a rare and severe infection characterized by hepatic parenchymal emphysema without liquefied abscess.^[1]^ It usually deteriorates quickly, resulting in death. Most patients had diabetes or a history of digestive system cancer.^[1–6]^ Computed tomography (CT) images are usually essential for diagnosing EH.^[7,8]^ To date, only a few cases have been reported since Blachar et al^[1]^ reported the first case in 2001. Unfortunately, most patients died within a short time because of septicemia or multiple organ failure.^[1–3,9]^ Our case report describes a relatively young man with diabetes who recovered from EH through a series of effective treatments.

## 2. Case report

The local ethics committee approved the study protocol. Written informed consent was obtained from the patient for the publication of this anonymized case. A 48-year-old man presented to the emergency department with nausea, vomiting (gastric contents) and diarrhea (loose and liquid stools) for 4 days. The patient had a history of poorly controlled diabetes mellitus and hypertension. Physical examination showed that the patient had a fever and was in distress.

Upon admission, laboratory examinations revealed blood glucose of 23.7mmol/L (3.9–6.1), glycosylated hemoglobin of 12.8% (4.0–6.0), white blood cell count of 12.9 × 10^9^/L (3.5–9.5), C-reactive protein of >200 mg/L (0.0–8.2), procalcitonin of 25.66 ng/mL (0.0–0.50), Na of 117.1 mmol/L (135.0–145.0), Cl of 84 mmol/L (95.0–105.0) and accu troponin 115.4 ng/L (<14.0). The liver profiles changed: glutamic oxaloacetic transaminase of 73.5 U/L (15.0–40.0), glutamic pyruvic transaminase of 60.9 U/L (9.0–50.0), alkaline phosphatase of 156 U/L (45.0–125.0), and lactate dehydrogenase 248 U/L (135.0–220.0).

**Figure 1.**
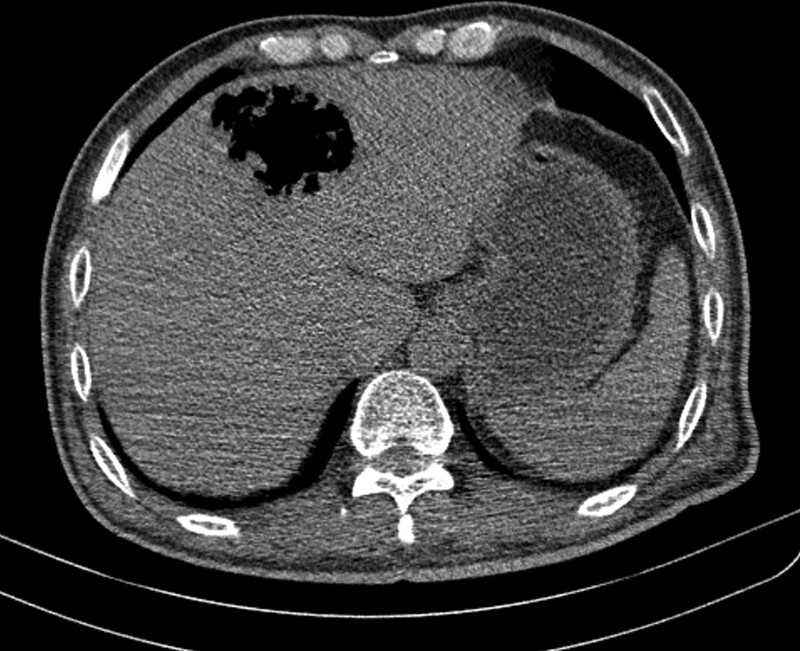
Initial computed tomography showed a large scale of air collection in the left liver lobe without fluid.

Plain abdominal CT was performed as soon as he visited the emergency department. It showed a 6.6 × 5.5 × 5.3 cm air collection with some remaining parenchyma debris in the left liver lobe, mainly in segments IV and II. There was no apparent fluid inside the lesion (Fig. 1). Abdominal CT features and laboratory examination results rationalized the diagnosis of EH.

**Figure 2.**
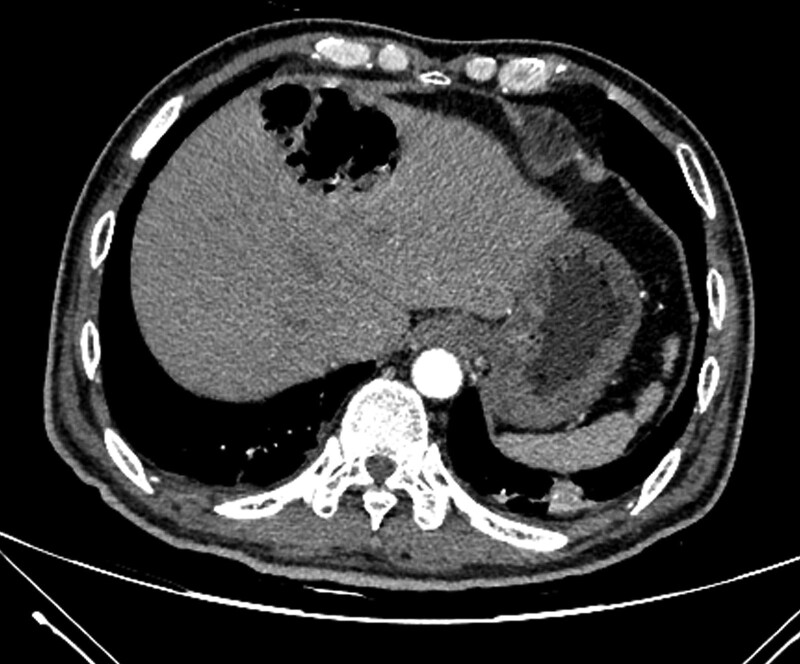
Enhanced abdominal computed tomography 2 days later showed the emphysematous alveolarization of liver parenchyma with some fluid collection around the lesion.

**Figure 3.**
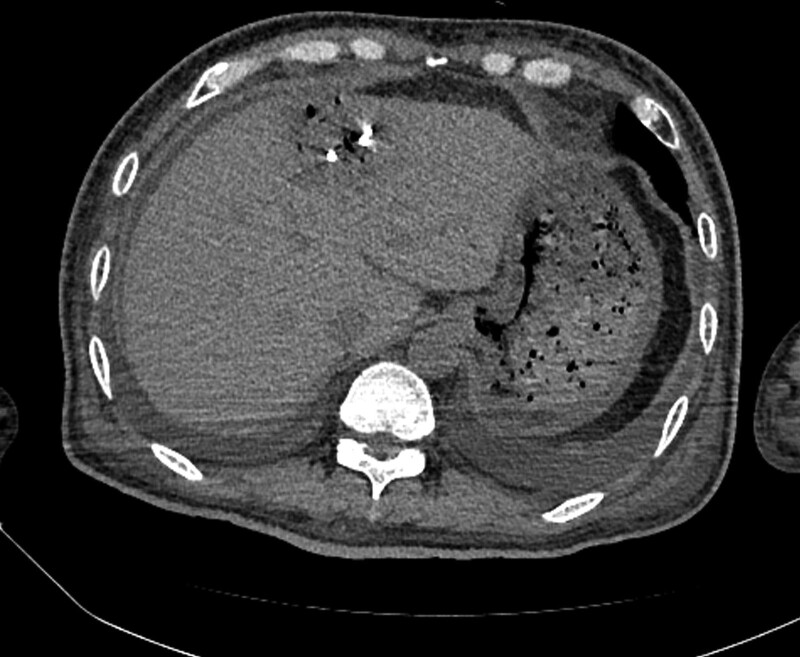
Plain computed tomography 6 days after the initial examination showed an apparent reduction of lesion range and air collection after percutaneous catheter drainage.

We controlled glucose levels strictly throughout the whole range of treatment. After using broad-spectrum antibiotics (ceftriaxone sodium) for approximately 2 days, the blood culture revealed an abundance of gram-negative bacilli, *Klebsiella oxytoca*, and the patient was commenced on sensitive antibiotics, cefoperazone, and sulbactam. We performed an enhanced abdominal CT 2 days after the initial CT scan. The extent of the lesion did not change and emphysematous alveolarization of the liver parenchyma was still apparent. Small amounts of pus and fluid were collected around the lesion (Fig. 2). Due to air, pus collection, and septicopyemia, we performed ultrasound-guided percutaneous catheter drainage, which drained a large amount of gas with little pus and blood. A follow-up unenhanced abdominal CT was performed 6 days after the first examination, and revealed a significant reduction of the lesion (Fig. 3). After strict glucose control, active antibiotics, percutaneous drainage, other symptomatic relief and supportive treatments, the patient was discharged 13 days after initial presentation. The patient was followed up for 2 months, and EH did not recur.

## 3. Discussion

EH is a necrotic hepatitis with hepatic parenchyma replaced by gas collection and no evidence of liquid.^[1]^ Though aggressive anti-infection therapy, most patients died soon because EH progressed rapidly. Only 3 female patients survived EH through active antibiotic treatment and percutaneous drainage with or without surgery (Table 1).^[4,7,8]^ In this case report, the patient was successfully treated with strict glycemic control, aggressive anti-infection treatment, and timely percutaneous drainage.

**Table 1:** Case reports of emphysematous hepatitis with successful treatments.

Ref.	Age/sex	Imaging	Treatment	Pathogen(s)	Outcome
Ghosn et al,^[4]^ 2019	38/F	CT: mixed collection containing air and debris (8 × 7 × 5.5 cm)	Urgent exploratory laparotomy; antibiotics	*Escherichia coli* and *Enterococcus faecium*	Survived
Estébanez-Ferrero et al,^[7]^ 2021	67/F	CT: abundant air in the hepatic parenchyma of segments VI, VII and VIII	Urgent surgery: drainage, debridement and lavage; antibiotics	*E. coli*	Survived
Francois et al,^[8]^ 2022	70/F	CT: air-filled cavity in the right liver lobe (9 cm)	Antibiotics; drainage	*E. coli, Streptococcus anginosus*, and *Klebsiella oxytoca*	Survived
This case	48/M	CT: air collection with some remaining parenchyma debris in the left liver lobe (6.6 × 5.5 × 5.3cm)	Antibiotics; drainage	*K. oxytoca*	Survived

CT = computed tomography.

EH affects women more than men, with a large age range from 38 to 80.^[2,4,10]^ It is a necrotizing infection of the hepatic parenchyma caused by *Escherichia coli* and *Enterococcus faecium, K. oxytoca, Enterobacter, Pseudomonas, Streptococcus* and so on.^[1,7]^ The patient in our case was infected with *K. oxytoca*. Many previous cases and our case revealed that diabetes mellitus seemed to be a major risk factor for EH.^[1,3,4,8,10,11]^ Gas collection in the lesion mainly comes from the mixed acid fermentation of glucose, including nitrogen, hydrogen, carbon dioxide, and oxygen.^[12]^ Patients with diabetes have elevated blood glucose levels, and associated microangiopathy impairs the transportation of gas.^[3]^ On the other hand, a history of digestive system cancer with or without surgery predisposes people to EH.^[2,5,6,13]^ The probable reason is that bile reflux could worsen the liver infection. Our patient was diabetic but did not have a history of digestive system cancer or surgery.

Patients with EH usually present to the hospital with primary symptoms of abdominal pain, vomiting, fever, and even changes in mental status. Imaging features, mainly the CT findings show extensive liver parenchyma necrosis substituted by gas and no evidence of fluid around the infected area.^[1]^ We should clearly specify that the gas exists in liver parenchyma. EH is different from pyogenic liver abscess with gas collection, whose imaging characteristics include mass effect with central low attenuation, annular enhancement, and edema of the adjacent liver parenchyma.^[14]^ In our case, the extent of EH was restricted and pus appeared after the use of sensitive antibiotics. EH seemed to develop into a pyogenic liver abscess and avoided further deterioration. Similar to our case, Ghosn et al^[4]^ reported a case of co-existence of EH and pyogenic liver abscess, confirmed by surgical exploration. Gas within the liver parenchyma also appears in hepatic infarcts and gas gangrene infection after liver transplantation with hepatic artery thrombosis.^[15]^

Once EH is diagnosed, patients should be treated with urgent surgical debridement. Two of the 3 surviving patients underwent urgent exploratory laparotomy (Table 1).^[4,7]^ Whether percutaneous catheter drainage is valid is controversial. Some authors pointed out that percutaneous catheter drainage was ineffective because there was no fluid or pus.^[6,7]^ However, a case of a 70-year-old woman survived EH through percutaneous pigtail catheter drainage without surgery (Table 1).^[8]^ Another 67-year-old female was discharged after urgent surgery and percutaneous drainage (Table 1).^[7]^ Therefore, percutaneous catheter drainage is necessary and may strengthen the effect of surgical debridement. Glucose-lowering treatment and aggressive combined antibiotic treatment are also necessary.^[7,8]^ After admission, we actively regulated the blood glucose levels and achieved results. The random blood glucose level was 23.7 mmol/L at the beginning and reduced to 9.4 mmol/L the day he was discharged from the hospital. Similarly to other surviving patients, our patient was commenced on sensitive antibiotics timely based on blood culture results. Strict glucose control and sensitive antibiotic treatment made him response to percutaneous drainage and survive the frequently fulminant EH.

In conclusion, we presented a rare case of EH with diabetes mellitus that was successfully treated. This case will strengthen the diagnostic capacity of EH and provide advice for its treatment. Effective therapeutic methods include urgent surgical debridement, percutaneous catheter drainage, antibiotic treatment, and strict glucose control.

## Author contributions

**Conceptualization:** Nannan Pan, Shuo Wang, Zhenwei Miao.

**Data curation:** Nannan Pan, Shuo Wang.

**Formal analysis:** Nannan Pan, Shuo Wang, Zhenwei Miao.

**Investigation:** Nannan Pan, Shuo Wang.

**Methodology:** Nannan Pan, Zhenwei Miao.

**Writing – original draft:** Nannan Pan, Shuo Wang.

**Writing – review & editing:** Zhenwei Miao.
